# Immunogenicity and safety of a single dose of adjuvanted RSVPreF3 vaccine in adults ≥60 years of age in China: results from a phase 3 immunobridging trial

**DOI:** 10.3389/fimmu.2026.1894773

**Published:** 2026-09-03

**Authors:** Xiang Guo, Guijun Ning, Dan Bi, Silvia Damaso, Monique Dodet, Fang Huang, Xinji Zhang, Flavio Genco, Hongmei Lu, Jingjing Zhao, Lili Jiang, Xiaoting Wu, Xiaojun Li, Laibao Yang, Nancy Dezutter, Xiaodong Sun, Jieqiong Hu

**Affiliations:** 1 Shanghai Municipal Center for Disease Control and Prevention, Shanghai, China; 2 GSK, Beijing, China; 3 GSK, Shanghai, China; 4 GSK, Wavre, Belgium; 5 GSK, Rixensart, Belgium; 6 Songjiang District Center for Disease Control and Prevention (Songjiang District Health Supervision Institute), Shanghai, China; 7 Jiading District Center for Disease Control and Prevention (Jiading District Health Supervision Institute), Shanghai, China; 8 Putuo District Center for Disease Control and Prevention (Putuo District Health Supervision Institute), Shanghai, China; 9 Minhang District Center for Disease Control and Prevention (Minhang District Health Supervision Institute), Shanghai, China; 10 Baoshan District Center for Disease Control and Prevention (Baoshan District Health Supervision Institute), Shanghai, China; 11 Pudong New Area Center for Disease Control and Prevention (Pudong New Area Health Supervision Institute), Shanghai, China

**Keywords:** adjuvanted RSVPreF3, Chinese adults, clinical trial, immunogenicity, RSV, safety, aged ≥60 years

## Abstract

**Introduction:**

Respiratory syncytial virus (RSV) is an important cause of severe respiratory disease, yet no vaccine is available for older adults in China. This phase 3, randomized, partially blind immunobridging study (NCT06551181) compared a single dose of adjuvanted RSVPreF3 vaccine in ≥60-year-old Chinese adults with a non-Chinese population.

**Methods:**

Chinese participants were randomized 2:1 to receive adjuvanted RSVPreF3 (RSVPreF3_China group) or placebo (Placebo_China); participants from six additional countries (RSVPreF3_Overseas) received adjuvanted RSVPreF3. The primary objective was to demonstrate non-inferiority of humoral immune responses (RSV-A/RSV-B neutralizing titers) at 1 month post-vaccination in Chinese versus non-Chinese participants. Non-inferiority was met if the upper limit of the 95% confidence interval (CI) was ≤1.5 for the geometric mean titer (GMT) ratio (RSVPreF3_Overseas over RSVPreF3_China) and ≤10% for the seroresponse rate (SRR) difference (RSVPreF3_Overseas minus RSVPreF3_China). Reactogenicity and safety were also assessed.

**Results:**

Overall, 2620 participants were exposed (RSVPreF3_China: 1199; Placebo_China: 601; RSVPreF3_Overseas: 820); 2522 were included in the per protocol set at 1 month post-vaccination (RSVPreF3_China: 1175; Placebo_China: 590; RSVPreF3_Overseas: 757). Non-inferiority criteria were met for both RSV-A and RSV-B, with 95% CI upper limits of 1.20 and 1.10 for GMT ratios and 3.40% and 4.89% for SRR differences, respectively. Neutralizing titers increased at 1 month in vaccine recipients: RSV-A/RSV-B GMTs were 5523.2/8073.2 ED60 (mean geometric increase [MGI]=7.89/6.40) in the RSVPreF3_China group and 6604.5/8286.2 ED60 (MGI=8.04/6.57) in the RSVPreF3_Overseas group; GMTs remained above baseline at 6 months post-vaccination. Solicited events were more frequent in vaccine- than placebo-Chinese recipients but were mostly mild-to-moderate and transient. Pain (in 41.7% of participants in the RSVPreF3_China group versus 4.2% in the Placebo_China group) and fatigue (13.6% versus 3.2%) were the most frequently reported administration-site and systemic events, respectively. The incidence of unsolicited adverse events (reported in 11.8%–13.8% of participants across Chinese groups), serious adverse events (3.4%–6.5% across all groups), and potential immune-mediated diseases (0.2%–0.3% across all groups) was low and comparable between groups.

**Conclusion:**

A single dose of adjuvanted RSVPreF3 was immunogenic and well tolerated in Chinese adults aged ≥60 years, supporting the extrapolation of global efficacy and a favorable benefit-risk profile in this population.

## 1 Introduction

Respiratory syncytial virus (RSV) remains a significant yet often underestimated cause of acute respiratory illness (ARI) and lower respiratory tract disease (LRTD) in young children (1), older adults (2, 3), and individuals with certain underlying conditions (4). Older adults, in particular those with comorbidities, have a higher risk of developing severe RSV-associated illness, leading to high hospitalization and mortality rates.

In China, RSV was identified as a leading cause of ARI between 2009 and 2019, accounting for 16.8% of ARI cases in individuals of all ages and 7.4% of those in adults ≥60 years of age (5). However, most available data on the burden of RSV-associated disease are focused on children <5 years of age, while little information is available on the burden of RSV among older Chinese adults.

Recently, several preventive options have become available for RSV-associated illness in older adult populations (6), including 3 vaccines. RSV vaccination has shown effectiveness against RSV-associated hospitalization, with estimates of 75% during the first season after vaccination (7) and 58% across 2 RSV seasons (8) among adults ≥60 years of age (including immunocompromised adults). Two of these vaccines, adjuvanted RSVPreF3 (*Arexvy*, GSK) and RSVpreF (*Abrysvo*, Pfizer), are also approved for use in adults aged ≥60 years in Macau and Hong Kong, but remain unavailable in mainland China (9).

Adjuvanted RSVPreF3 is an AS01_E_-adjuvanted RSV prefusion F protein–based vaccine that was assessed in a large phase 3 clinical trial (AReSVi-006) in adults ≥60 years of age (10–12). The vaccine was shown to be immunogenic, well tolerated, and efficacious against RSV-LRTD across 3 consecutive RSV seasons. Although conducted in 17 countries across both hemispheres, the trial did not include participants from mainland China.

**Figure 1:**
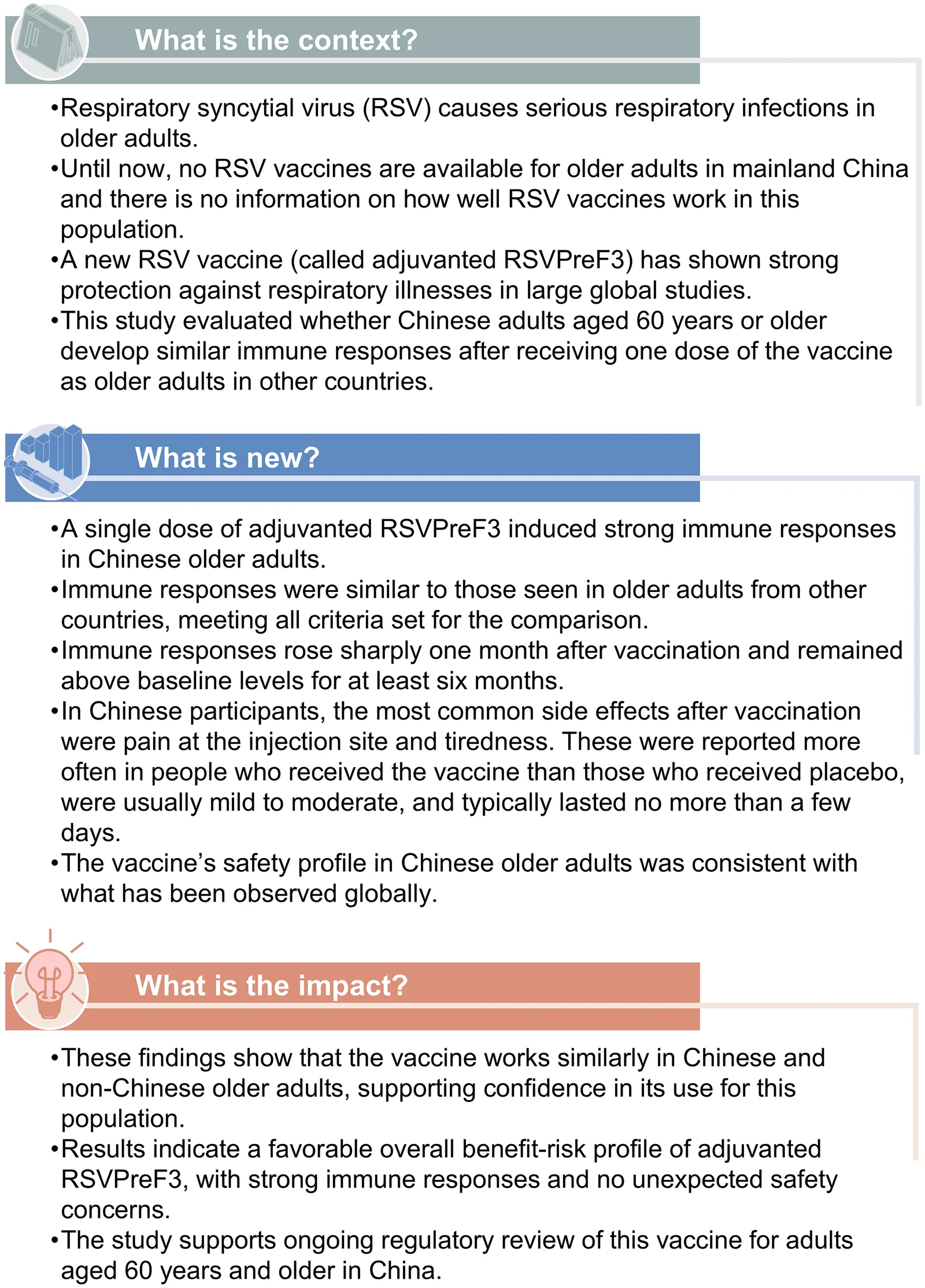
Plain language summary.

Here, we report the immunogenicity of adjuvanted RSVPreF3 in the first completed phase 3 clinical trial evaluating the vaccine in adults aged ≥60 years in China. Non-Chinese participants from countries where the global efficacy trial AReSVi-006 was conducted were also enrolled in this study for immunogenicity and safety comparisons, while vaccine reactogenicity and the occurrence of ARI were assessed among Chinese participants. Findings are summarized in plain language in **Figure 1**.

## 2 Methods

### 2.1 Study design and participants

This phase 3, randomized, controlled, partially blind, immunobridging study was conducted in 12 centers in China and 33 centers in 6 other countries (Finland, Japan, Poland, Republic of Korea, Spain, and the United Kingdom), between 5 August 2024 and 27 November 2025.

Adults ≥60 years of age living in community dwellings, who were medically stable as assessed by the investigator, had not previously received any RSV vaccine, and who provided written or witnessed informed consent were enrolled in the study. Individuals with chronic medical conditions, such as diabetes, hypertension, or cardiac disease (treated or untreated), were eligible if the investigator considered the participant’s condition to be medically stable. Inclusion and exclusion criteria are detailed in the **Supplementary Material, Text S1**. Enrolment in the study was performed throughout the year and was not restricted to periods corresponding to the RSV season in each country.

Participants from China were randomized 2:1 to receive either 1 dose of adjuvanted RSVPreF3 (RSVPreF3_China group) or placebo (Placebo_China group) on Day 1. Participants from China were randomly allocated to each group using an interactive web response system. Participants from the other countries received 1 dose of adjuvanted RSVPreF3 (RSVPreF3_Overseas group). The study was observer-blind for participants in China, with participants and all personnel involved in clinical evaluation and laboratory testing remaining blinded to treatment assignment. Vaccines were prepared and administered by unblinded staff not involved in assessments or endpoint evaluation. The RSVPreF3_Overseas arm was open-label.

The study documents were approved by institutional review boards/independent ethics committees in each country. The trial was conducted in accordance with Good Clinical Practice and the Declaration of Helsinki and is registered with ClinicalTrials.gov on 9 August 2024 (NCT06551181). The study protocol is available at https://www.gsk-studyregister.com/trials/219815.

### 2.2 Objectives

The co-primary objectives were to demonstrate the non-inferiority of humoral immune responses (RSV-A/RSV-B neutralization titers) in Chinese adults ≥60 years of age in the RSVPreF3_China group compared to those in the RSVPreF3_Overseas group at 1 month after vaccination with adjuvanted RSVPreF3. Non-inferiority was demonstrated if the upper limit (UL) of the 2-sided 95% confidence interval (CI) for the geometric mean titer (GMT) ratio (RSVPreF3_Overseas group over RSVPreF3_China group) was ≤1.5 and the UL of the 2-sided 95% CI for the seroresponse rate (SRR) difference (RSVPreF3_Overseas group minus RSVPreF3_China group) was ≤10% for both RSV-A and RSV-B.

Secondary objectives included the evaluation of humoral immune responses in Chinese versus non-Chinese participants enrolled in the study up to 6 months post-vaccination. Safety and reactogenicity were also assessed. Throughout the study, the occurrence, duration, symptoms/signs, and severity of polymerase chain reaction (PCR)-confirmed RSV-ARI/LRTD episodes were evaluated in Chinese participants only.

### 2.3 Study procedures

Adjuvanted RSVPreF3 (0.5 mL) or placebo (at least 0.5 mL; NaCl solution) were administered by intramuscular injection in the deltoid of the nondominant arm. Each dose of reconstituted adjuvanted RSVPreF3 contained 120 µg RSVPreF3 antigen and AS01_E_ adjuvant. AS01_E_ is an Adjuvant System containing 3-*O*-desacyl-4′-monophosphoryl lipid A (MPL), *Quillaja saponaria* Molina, fraction 21 (QS-21, licensed by GSK from Antigenics LLC, a wholly owned subsidiary of Agenus Inc., a Delaware, USA corporation), and liposome (25 µg MPL and 25 µg QS-21).

**Figure 2:**
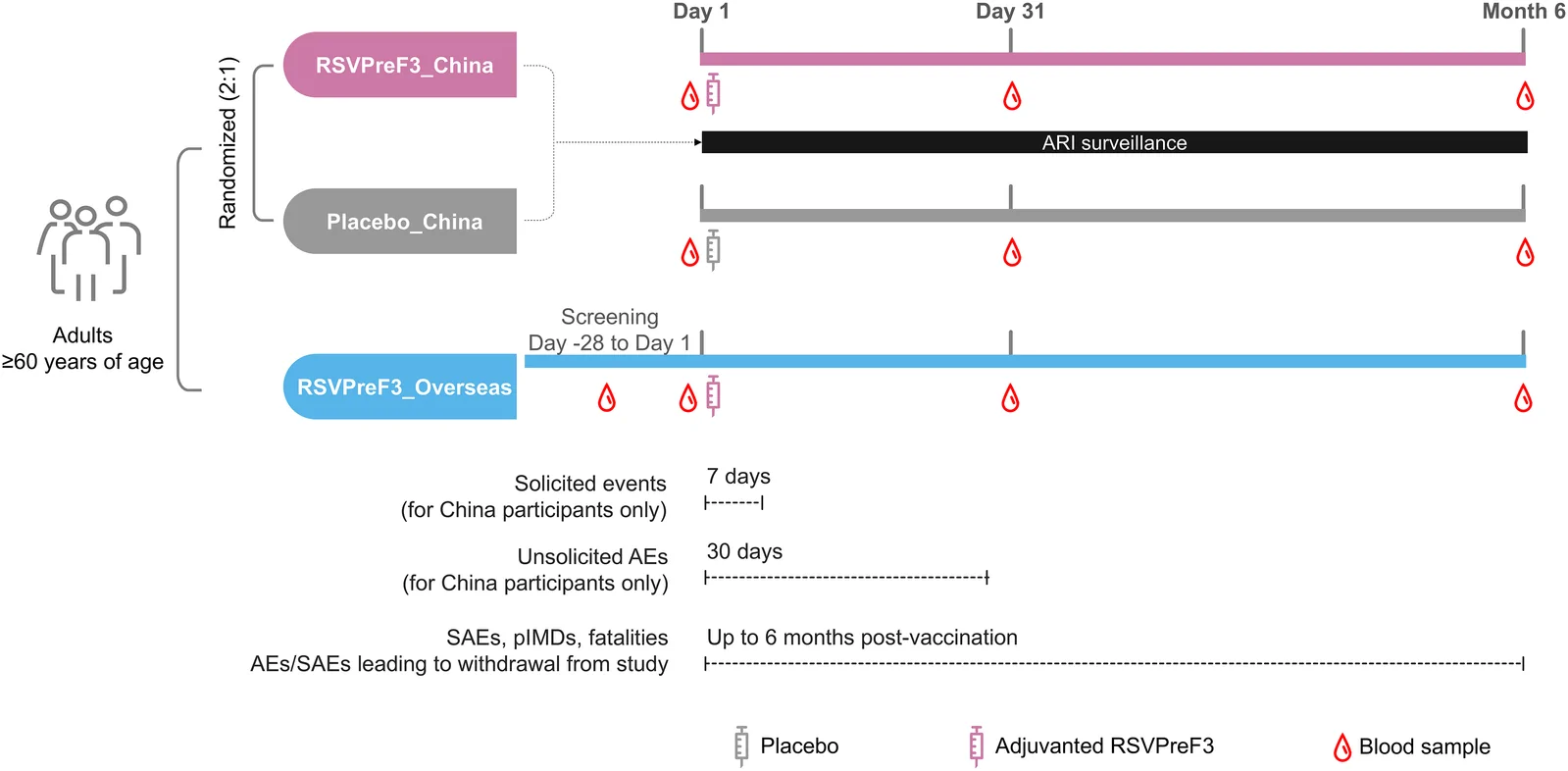
Study design. AE, adverse event; ARI, acute respiratory illness; pIMD, potential immune-mediated disease; SAE, serious AE.

Blood samples (~10 mL each) for immunogenicity assessments were collected from all participants pre-vaccination (Day 1), at 1 month (Day 31), and at 6 months post-vaccination (Month 6) (**Figure 2**). RSV-A and RSV-B neutralizing titers were assessed as described previously (10), and expressed as estimated dilution 60 (ED60), defined as the inverse of the interpolated serum dilution that yields a 60% reduction in the number of plaques compared to the virus control wells. In addition, ED60 neutralization titers were converted to concentrations in International Units (IU)/mL using secondary standards calibrated against the international reference (13, 14), which were included in each assay run to enable conversion. The SRR was defined as the proportion of participants having ≥4-fold increase in neutralizing titers (1 month post-vaccination over pre-vaccination), in accordance with the clinical development program of adjuvanted RSVPreF3 (15).

Solicited events were assessed in participants from China only, who used paper diary cards to record solicited administration-site and systemic events with onset within 7 days post-vaccination. All solicited events were graded by intensity by the investigator from mild (Grade 1) to severe (Grade 3). Grade 3 events were defined as significant pain at rest, preventing normal everyday activities for pain, a diameter >100 mm for swelling and erythema, an axillary temperature >39°C for fever, and as preventing normal everyday activities for the other events. The grading of solicited events was also done according to the guiding principles for grading criteria for adverse reactions in clinical trials of prophylactic vaccines formulated by the National Medical Products Administration (NMPA) in China (version 2019) (16). According to the Chinese grading scale, Grade 3 erythema or swelling was defined as a diameter ≥100 mm and Grade 3 fever, as an axillary temperature ≥38.5°C; additionally, Grade 4 fever (≥39.5°C lasting more than 3 days) was reported.

Unsolicited adverse events (AEs) were recorded by the investigator for 30 days post-vaccination for participants in China and graded by intensity. Serious AEs (SAEs), potential immune-mediated diseases (pIMDs), SAEs of atrial fibrillation, and (S)AEs leading to withdrawal were reported throughout the study in all groups. Investigators used their clinical judgment to decide whether AEs were related to study interventions.

Surveillance for detection of ARI episodes was carried out throughout the study in China, via spontaneous reporting by the participant (starting on Day 1) and by scheduled site staff contacts (starting from Day 31 onwards) with different frequencies of contact during the RSV season and the interseason periods. All participants reporting ≥2 ARI symptoms/signs meeting the ARI case definition (presence of ≥2 respiratory symptoms/signs for at least 24 hours or ≥1 respiratory symptom/sign and 1 systemic symptom/sign for at least 24 hours, as described in **Supplementary Table 1**) were followed-up for ARI assessment. Nasal self-swab or nasal and throat swab samples (by study staff) were collected and tested for RSV by reverse transcription PCR (RT-PCR). Case definitions for RSV-ARI/LRTD are detailed in **Supplementary Table 1**. Each ARI episode was diagnosed and managed according to local standard of care.

### 2.4 Statistical methods

The study was designed to enroll approximately 2600 participants, including 1800 participants in China, and 800 participants in the RSVPreF3_Overseas group. A sensitivity analysis was performed under multiple plausible immunogenicity scenarios, assuming a 10% attrition rate. The analysis demonstrated that the sample size would yield ≥90% power for evaluating the co-primary non-inferiority objectives. Power was estimated using a 2-sample t-test for GMT comparisons, and the Miettinen and Nurminen (17) method for SRRs.

Immunogenicity was assessed in the per protocol set that included all eligible participants who received the study intervention as per protocol, had immunogenicity results pre- and post-vaccination, complied with blood draw intervals, had no intercurrent conditions that could interfere with immunogenicity, and no prohibited concomitant medication/vaccination.

For the co-primary immunogenicity endpoints, GMT ratios with 2-sided 95% CIs were calculated with an ANCOVA model on log_10_-transformed titers for each neutralization titer; the model included the group as fixed effect and the baseline log_10_-transformed titer as covariate. The 2-sided 95% CIs for group SRR difference were derived using the Miettinen-Nurminen method (17). Primary analysis results were reported as ED60.

Within-group mean geometric increases (MGIs), defined as the geometric mean of the within-participant ratios of the post-vaccination over pre-vaccination titer, were also estimated. 95% CIs for GMTs and MGIs were based on a back-transformation of CIs for the mean of log_10_-transformed values. When computing GMTs, SRRs, MGIs, and between-group differences, titers below the assay cut-off (lower limit of quantification [LLOQ]) were replaced by half the assay cut-off (LLOQ/2) and titers above the upper limit of quantification (ULOQ) were replaced by the ULOQ. All analyses of between-group secondary immunogenicity endpoints were descriptive, with no adjustment for multiplicity.

Safety analyses were conducted in the exposed set, which included all participants receiving study intervention. The percentage of participants reporting AEs was calculated with exact 95% CIs using the Clopper-Pearson method (18).

For ARI surveillance, the number and percentage (with exact 95% CIs) of participants who reported at least 1 RSV-confirmed ARI/adjudicated LRTD episodes were computed.

Analyses were performed using SAS Viya version 4.0. Missing data were not imputed.

## 3 Results

### 3.1 Study participants

**Figure 3:**
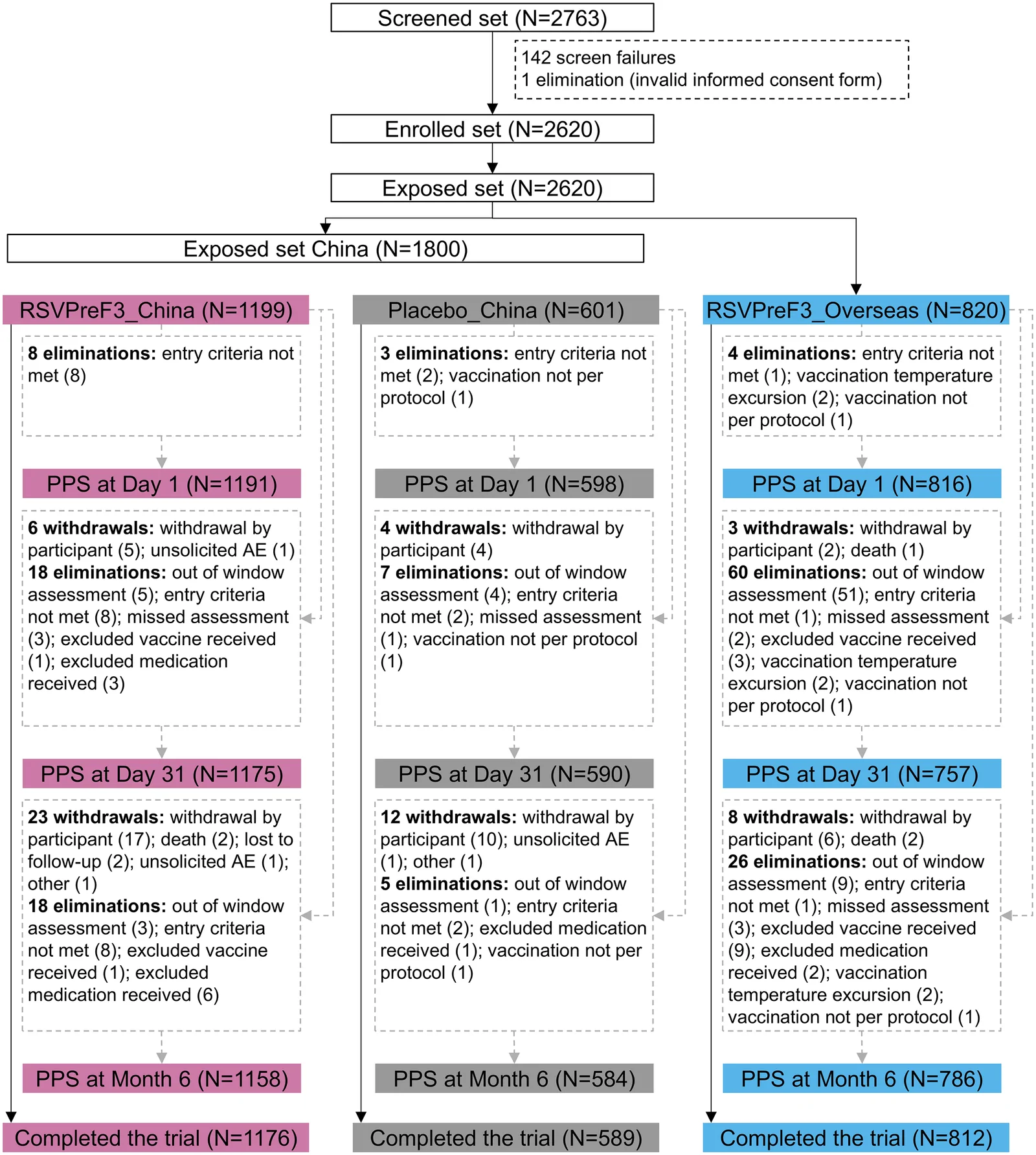
Study participant flowchart. AE, adverse event; N, number of participants in each group; PPS, per protocol set.

Overall, 2620 participants were enrolled and received the study intervention: 1199 in the RSVPreF3_China group, 601 in the Placebo_China group, and 820 in the RSVPreF3_Overseas group; 1191, 598, and 816 participants, respectively, were included in the per protocol set at Day 1 (**Figure 3**). In total, 2577 participants completed the study: 1176 in the RSVPreF3_China group, 589 in the Placebo_China group, and 812 in the RSVPreF3_Overseas group, with the most common reason for not completing being withdrawal by the participant (**Figure 3**).

In the per protocol set at Day 1, the mean age at vaccination was 70.1 years (varying from 69.8 to 70.5 across the 3 groups) and most participants (50.6% overall, 48.5%–52.3% across groups) were in the 60–69 years of age category. Slightly more than half of participants (52.0% overall, 50.3%–53.6% across groups) were female. Characteristics were balanced between the RSVPreF3_China and Placebo_China groups. Some differences were observed between Chinese and overseas participants for body mass index (≤25.1 versus 27.6 kg/m^2^), frailty status (≥49.8% versus 26.1% pre-frail and ≤49.3% versus 73.7% fit participants), and smoking status for tobacco (≥17.6% versus 10.8% current smokers) (**Table 1**). Baseline characteristics for the exposed set are detailed in **Supplementary Table 2**.

**Table 1:** Participant characteristics at baseline (per protocol set at day 1).

Characteristic	RSVPreF3_China N=1191	Placebo_China N=598	RSVPreF3_Overseas N=816	Total N=2605
Mean age ± SD, years	69.8 ± 6.6	69.9 ± 6.7	70.5 ± 6.8	70.1 ± 6.7
Age category, n (%)				
60–69 years	608 (51.0)	313 (52.3)	396 (48.5)	1317 (50.6)
70–79 years	476 (40.0)	221 (37.0)	322 (39.5)	1019 (39.1)
≥80 years	107 (9.0)	64 (10.7)	98 (12.0)	269 (10.3)
Female, n (%)	616 (51.7)	301 (50.3)	437 (53.6)	1354 (52.0)
Race, n (%)				
Asian - Central/South Asian Heritage	0 (0.0)	0 (0.0)	9 (1.1)	9 (0.3)
Asian - Chinese Heritage	1191 (100)	598 (100)	2 (0.2)	1791 (68.8)
Asian - Korean Heritage	0 (0.0)	0 (0.0)	60 (7.4)	60 (2.3)
Asian - Japanese Heritage	0 (0.0)	0 (0.0)	30 (3.7)	30 (1.2)
White-Caucasian/European Heritage	0 (0.0)	0 (0.0)	704 (86.3)	704 (27.0)
Other	0 (0.0)	0 (0.0)	11 (1.4)	11 (0.4)
Mean BMI (± SD), kg/m²	25.0 ± 3.3	25.1 ± 3.1	27.6 ± 4.8	25.9 ± 3.9
Frailty status, n (%)				
Frail	13 (1.1)	5 (0.8)	2 (0.2)	20 (0.8)
Pre-frail	607 (51.0)	298 (49.8)	213 (26.1)	1118 (42.9)
Fit	571 (47.9)	295 (49.3)	601 (73.7)	1467 (56.3)
Smoker status for tobacco				
Current smoker	210 (17.6)	110 (18.4)	88 (10.8)	408 (15.7)
Former smoker	167 (14.0)	80 (13.4)	316 (38.7)	563 (21.6)
Never smoked	814 (68.3)	408 (68.2)	412 (50.5)	1634 (62.7)
Smoker status for e-cigarettes				
Current smoker	0 (0.0)	0 (0.0)	0 (0.0)	0 (0.0)
Former smoker	0 (0.0)	0 (0.0)	5 (0.6)	5 (0.2)
Never smoked	1191 (100)	598 (100)	798 (97.8)	2587 (99.3)
Missing	0 (0.0)	0 (0.0)	13 (1.6)	13 (0.5)

BMI, body mass index; n (%), number (percentage) of participants in each category; N, number of participants in each group; SD, standard deviation.

Other included American Indian or Alaska Native, Asian - South East Asian Heritage, and Black or African American.

### 3.2 Immunogenicity

**Figure 4:**
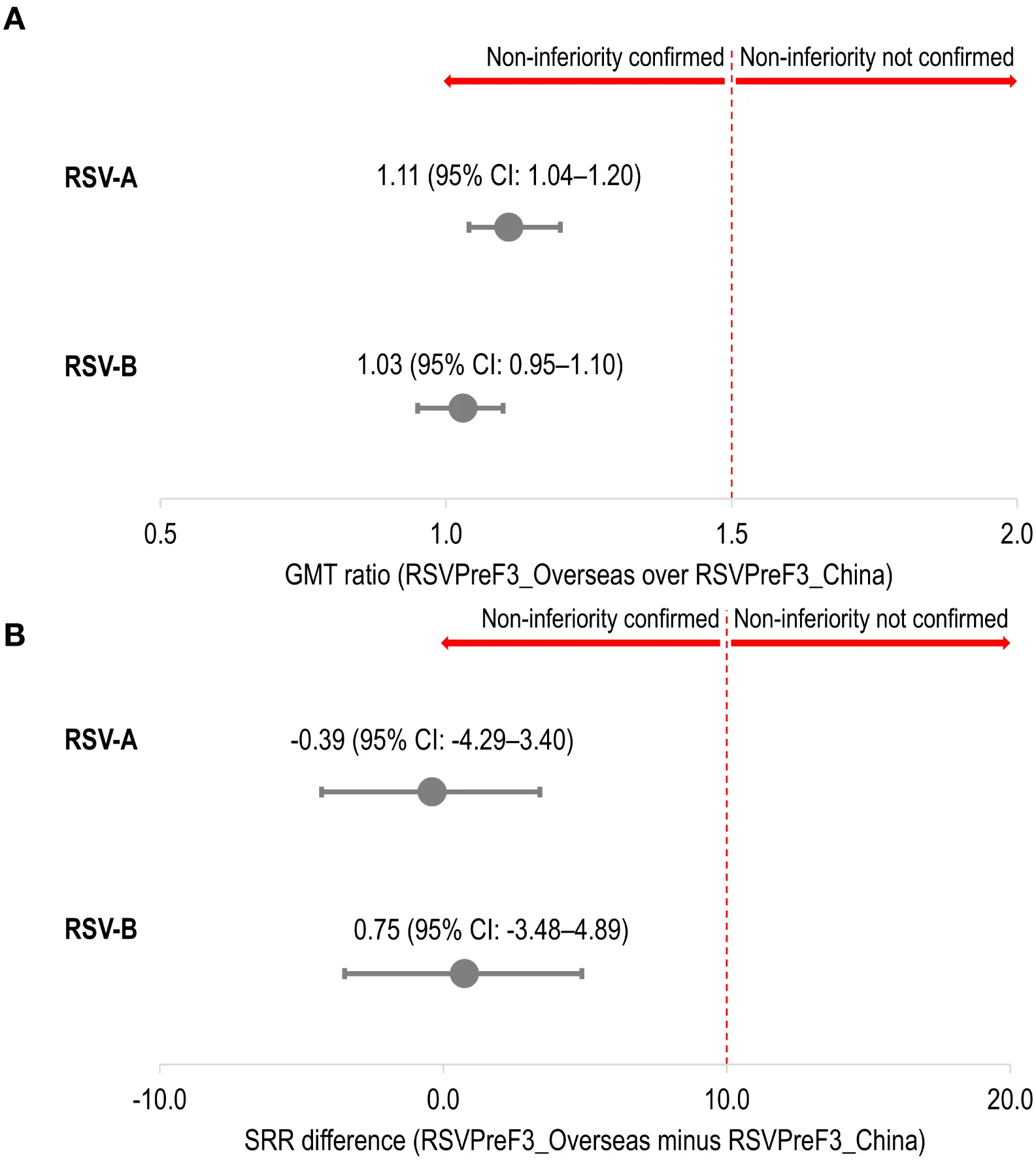
Summary of results of non-inferiority objectives for the GMT ratio **(A)** and the SRR difference **(B)** at 1 month post-vaccination (per protocol set). CI, confidence interval; GMT, geometric mean titer; RSV, respiratory syncytial virus; SRR, seroresponse rate. The SRR was defined as the proportion of participants having ≥4-fold increase in neutralizing titers (1 month post-vaccination over pre-vaccination).

The per protocol set at Day 31 included 2522 participants: 1175 in the RSVPreF3_China group, 590 in the Placebo_China group, and 757 in the RSVPreF3_Overseas group. The adjusted GMT ratio for neutralizing titers (RSVPreF3_Overseas group over RSVPreF3_China group) was 1.11 (95% CI: 1.04–1.20) for RSV-A and 1.03 (95% CI: 0.95–1.10) for RSV-B. The SRR difference (RSVPreF3_Overseas group minus RSVPreF3_China group) was -0.39 (95% CI: -4.29–3.40) for RSV-A and 0.75 (95% CI: -3.48–4.89) for RSV-B. Non-inferiority was demonstrated for all co-primary objectives: at 1 month post-vaccination, the UL of the 2-sided 95% CI for the adjusted GMT ratio was <1.5, and the UL of the 2-sided 95% CI for the SRR difference was <10% for both RSV-A and RSV-B (**Figure 4**, **Supplementary Table 3**). Similar observations were made when neutralizing concentrations (IU/mL) were used (**Supplementary Table 4**). Analyses performed on the exposed set confirmed the findings of the primary analyses in the per protocol set (**Supplementary Table 5**).

**Figure 5:**
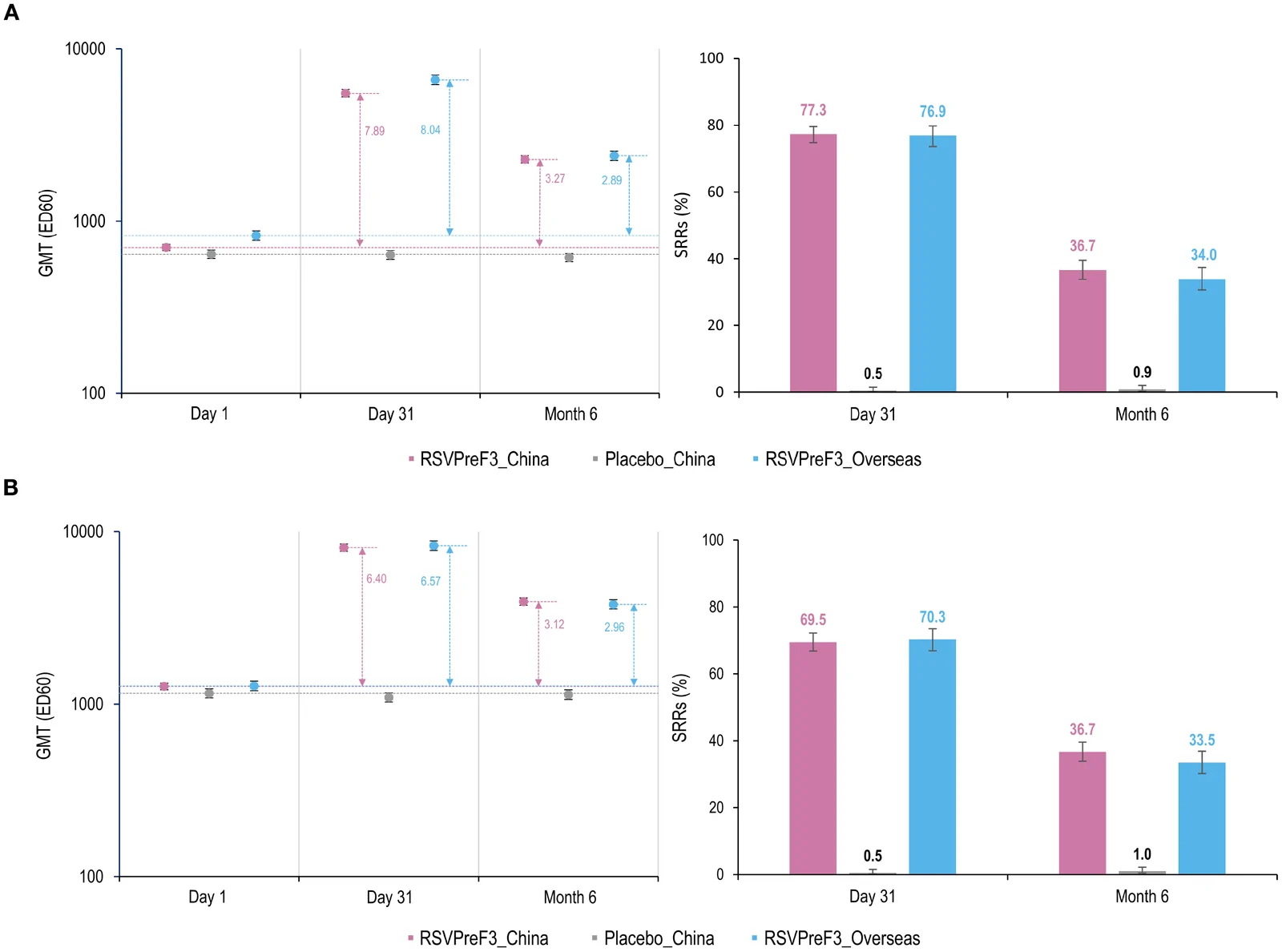
Humoral immune responses for RSV-A **(A)** and RSV-B **(B)** in the Chinese and non-Chinese study population (per protocol set). CI, confidence interval; ED60, estimated dilution 60; GMT, geometric mean titer; SRR, seroresponse rate. The SRR was defined as the proportion of participants having ≥4-fold increase in neutralizing titers (1 month post-vaccination over pre-vaccination). In the left panels, values indicate mean geometric increases from pre-vaccination. In the right panels, values show the SRR. Error bars indicate 95% CIs.

All participants had detectable RSV-A/RSV-B neutralizing titers at baseline. One month post-vaccination, RSV-A neutralizing antibody titers increased markedly in both the RSVPreF3_China and the RSVPreF3_Overseas groups, while no change was observed in the Placebo_China group. In the RSVPreF3_China group, GMTs increased from 703.3 to 5523.2 ED60, with an MGI of 7.89. In the RSVPreF3_Overseas group, GMTs rose from 825.8 to 6604.5 ED60, with an MGI of 8.04. A ≥4-fold rise at 1 month was observed in 77.3% of participants in the RSVPreF3_China group, 76.9% in the RSVPreF3_Overseas group, and 0.5% in the Placebo_China group. Six months post-vaccination, RSV-A neutralizing antibody titers decreased in both the RSVPreF3_China (2283.4 ED60; MGI of 3.27) and the RSVPreF3_Overseas (2395.6 ED60; MGI of 2.89) groups but remained over the baseline levels. SRRs were 36.7%, 34.0%, and 0.9% in the RSVPreF3_China, RSVPreF3_Overseas, and Placebo_China groups, respectively (**Figure 5A**, **Supplementary Table 6**).

One month post-vaccination, RSV-B neutralizing antibody titers increased in both the RSVPreF3_China (from 1264.9 to 8073.2 ED60; MGI of 6.40) and the RSVPreF3_Overseas (from 1274.2 to 8286.2 ED60; MGI of 6.57) groups; no change was observed in the Placebo_China group. SRRs were 69.5%, 70.3%, and 0.5%, respectively. Six months post-vaccination, RSV-B neutralizing antibody titers decreased to 3931.5 ED60 in the RSVPreF3_China group and 3785.0 ED60 in the RSVPreF3_Overseas groups but remained above the baseline levels (MGIs of 3.12 and 2.96, respectively). SRRs were 36.7%, 33.5%, and 1.0% in the RSVPreF3_China, RSVPreF3_Overseas, and Placebo_China groups, respectively (**Figure 5B**, **Supplementary Table 6**). Similar observations were made when neutralizing concentrations (IU/mL) were used (**Supplementary Table 6**).

### 3.3 Safety

**Figure 6:**
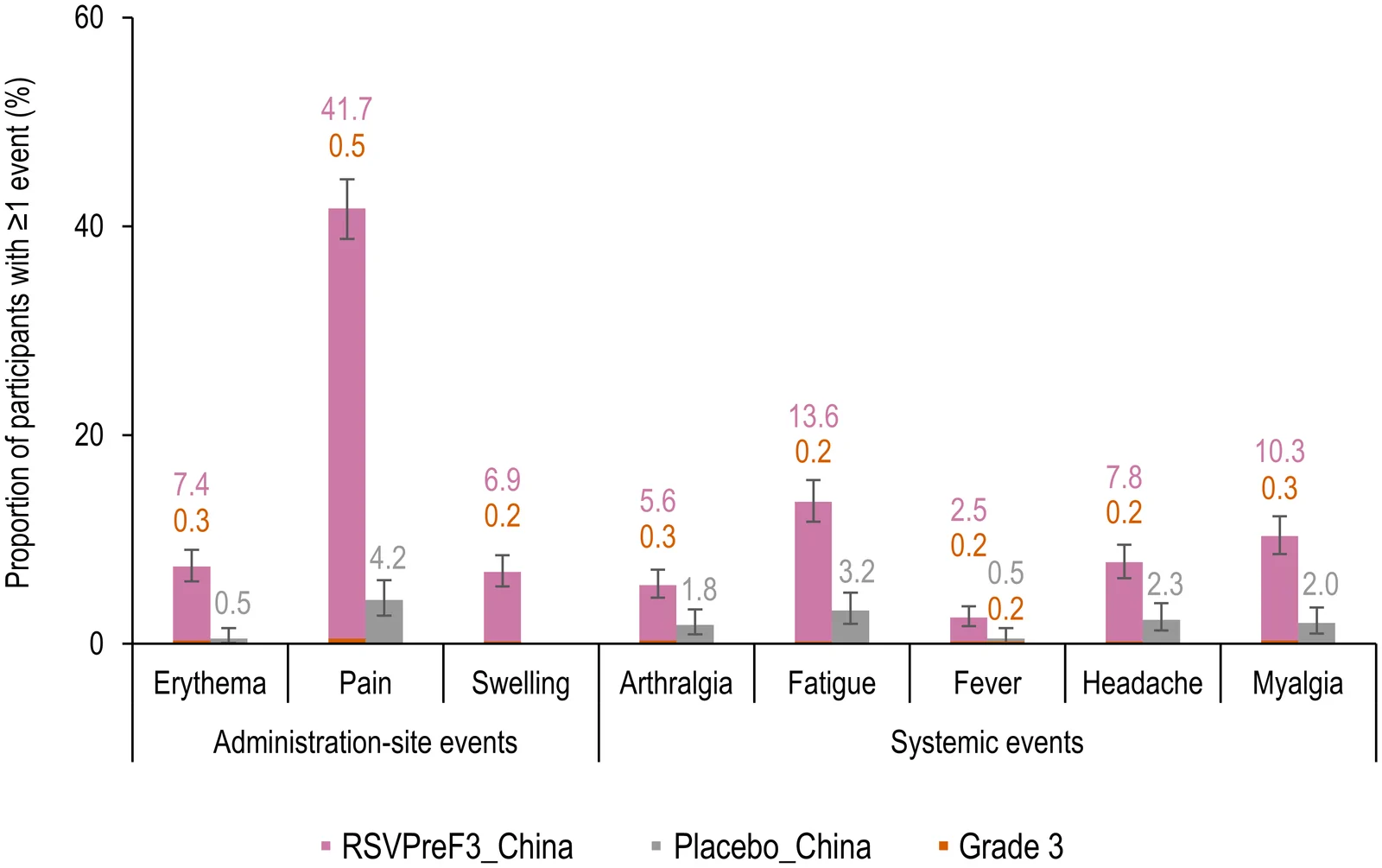
Percentages of Chinese participants reporting at least 1 solicited event within 7 days post-vaccination (exposed set). Values above bars show the percentage of participants reporting ≥1 event of any intensity (group color) and of Grade 3 intensity (dark orange color). Fever was defined as an axillary temperature ≥38˚C. Grade 3 intensity was defined as diameter >100 mm for erythema and swelling, pain at rest, preventing normal everyday activities for pain, temperature >39˚C for fever, and preventing normal everyday activities for all other solicited events. Error bars indicate 95% confidence intervals.

Among Chinese participants, a higher reporting rate of solicited events was seen in the RSVPreF3_China group as compared with the Placebo_China group. In both groups, the most frequently reported solicited administration-site event was pain, in 41.7% of adjuvanted RSVPreF3-vaccinated participants and 4.2% of placebo recipients. The most frequently reported solicited systemic event was fatigue (13.6% of participants in the RSVPreF3_China group and 3.2% of participants in the Placebo_China group). Grade 3 administration-site events were reported by 0.2% (for swelling) to 0.5% (for pain) of participants in the RSVPreF3_China group and none in the Placebo_China group. Grade 3 systemic events were reported by 0.2% (for fatigue, fever, and headache) to 0.3% (for arthralgia and myalgia) of participants in the RSVPreF3_China group and 0.2% (for fever) of participants in the Placebo_China group (**Figure 6**, **Supplementary Table 7**). The median duration of each solicited event was ≤3.0 days for both administration-site and systemic events, regardless of intensity. When classified according to the China NMPA scale, there was no difference in the frequency of Grade 3 solicited erythema or swelling, and Grade 3 fever occurred at similar frequencies; no Grade 4 fever was reported (**Supplementary Table 7**).

In Chinese participants, within 30 days from vaccination, unsolicited AEs were reported by 13.8% of participants in the RSVPreF3_China group and 11.8% in the Placebo_China group, with AEs considered related to vaccination for 2.8% and 2.2% of participants, respectively. Grade 3 unsolicited events were reported by 1.1% and 0.8% of participants in the RSVPreF3_China group and Placebo_China group, respectively, and 9.1% and 8.2% of participants, respectively, reported at least 1 medically attended unsolicited AE (**Table 2**).

**Table 2:** Safety summary (exposed set).

AE	RSVPreF3_China (N = 1199)		Placebo_China (N = 601)		RSVPreF3_Overseas (N = 820)	
	n	% (95% CI)	n	% (95% CI)	n	% (95% CI)
Unsolicited AEs	165	13.8 (11.9–15.8)	71	11.8 (9.3–14.7)		NC
Grade 3	13	1.1 (0.6–1.8)	5	0.8 (0.3–1.9)		NC
Related	34	2.8 (2.0–3.9)	13	2.2 (1.2–3.7)		NC
Grade 3, related to study intervention	1	0.1 (0.0–0.5)	2	0.3 (0.0–1.2)		NC
Medically attended	109	9.1 (7.5–10.9)	49	8.2 (6.1–10.6)		NC
Fatal events	2	0.2 (0.0–0.6)	0	0.0 (0.0–0.6)	2	0.2 (0.0–0.9)
SAEs	53	4.4 (3.3–5.7)	39	6.5 (4.7–8.8)	28	3.4 (2.3–4.9)
Related to study intervention	2	0.2 (0.0–0.6)	2	0.3 (0.0–1.2)	0	0.0 (0.0–0.4)
pIMDs	3	0.3 (0.1–0.7)	1	0.2 (0.0–0.9)	2	0.2 (0.0–0.9)
Related	0	0.0 (0.0–0.3)	1	0.2 (0.0–0.9)	0	0.0 (0.0–0.4)
Atrial fibrillation reported as unsolicited AE	1	0.1 (0.0–0.5)	0	0.0 (0.0–0.6)		NC
Atrial fibrillation reported as SAE	1	0.1 (0.0–0.5)	1	0.2 (0.0–0.9)	1	0.1 (0.0–0.7)

AE, adverse event; CI, confidence interval; n (%), number (percentage) of participants with at least 1 AE; N, total number of participants; NC, not collected; pIMD, potential immune-mediated disease; SAE, serious adverse event.

One participant in the RSVPreF3_China group discontinued the study due to an AE (inflammation) and 1 participant in the Placebo_China group discontinued the study due to an SAE (cerebral infarction). None of the events were considered as related to study intervention by the investigator.

Two participants in the RSVPreF3_China group and 2 in the RSVPreF3_Overseas group had fatal SAEs (**Table 2**). In the RSVPreF3_China group, the SAEs were arteriosclerosis coronary artery disease in one participant and bronchitis in another participant with arteriosclerosis coronary artery disease. In the RSVPreF3_Overseas group, SAEs included pneumonia in a participant with hypertensive heart disease and adenocarcinoma of colon in another participant. None of the events were considered related to the study intervention by the investigator.

At least 1 SAE with onset within 6 months from vaccination was reported in 53/1199 (4.4%) participants in the RSVPreF3_China group, 39/601 (6.5%) in the Placebo_China group, and 27/820 (3.3%) in the RSVPreF3_Overseas group; up to study end, an additional SAE occurred in the RSVPreF3_Overseas group (**Table 2**). Two SAEs were considered related to study intervention by the investigator in the RSVPreF3_China group: arthralgia in an 82-year-old male and segmental glomerulosclerosis in a 70-year-old female; both recovered/resolved by study end. Two other SAEs in participants in the Placebo_China group (gouty arthritis in a 75-year-old male and asthma in a 62-year-old male) were also considered related to study intervention by the investigator. All SAEs considered related to study intervention had onset within 6 months following vaccination.

pIMDs were reported in 0.2%–0.3% of participants across all groups (**Table 2**). One pIMD was considered related to the study intervention by the investigator, occurring in a participant in the Placebo_China group (gouty arthritis). No events of neurological demyelinating diseases, including Guillain-Barré syndrome and acute disseminated encephalomyelitis, were reported.

Atrial fibrillation was reported as an SAE in 1 participant each in the RSVPreF3_China, Placebo_China, and RSVPreF3_Overseas groups. In addition, 1 participant in the RSVPreF3_China group reported atrial fibrillation as a non-serious unsolicited AE within 30 days from vaccination. None of these events were considered by the investigator as related to the study intervention.

### 3.4 ARI surveillance

From Day 1 to study end, 173 (14.4%) participants in the RSVPreF3_China group and 76 (12.6%) participants in the Placebo_China group reported at least 1 ARI episode; episodes were confirmed as RSV-ARI in only 3 (0.5%) participants, all in the placebo group. At least 1 LRTD episode was reported by 34 (2.8%) participants in the RSVPreF3_China group and 19 (3.2%) participants in the Placebo_China group. Only 1 episode in 1 (0.2%) placebo recipient was confirmed as RSV-LRTD and was not severe.

## 4 Discussion

In this immunobridging study, a single dose of adjuvanted RSVPreF3 elicited similar humoral immune responses in Chinese adults ≥60 years of age and the global, non-Chinese population enrolled in the same study. Immunogenicity findings were also consistent with those observed in the AReSVi-006 pivotal trial (10), in which vaccine efficacy was demonstrated.

For both RSV-A and RSV-B, a similar longitudinal profile was observed in participants vaccinated with adjuvanted RSVPreF3, with a sharp increase in GMTs at 1 month post-vaccination, followed by a decline at 6 months post-vaccination. These observations are in line with results obtained for adjuvanted RSVPreF3 in other phase 3 clinical trials (12, 19, 20). At 1 month post-vaccination, humoral immune responses in Chinese participants were non-inferior to those observed in the non-Chinese population for both RSV-A and RSV-B, suggesting that ethnic or regional differences do not impact the vaccine’s immunogenicity. RSV-A and RSV-B GMTs and SRRs in Chinese participants were comparable to those observed in the population of the AReSVi-006 trial, in which a single vaccine dose showed an efficacy of 82.6% (96.95% CI: 57.9–94.1) (10), 67.2% (97.5% CI: 48.2–80.0) (11), and 62.9% (97.5% CI: 46.7–74.8) (12) against RSV-LRTD over 1, 2, and 3 consecutive RSV seasons, respectively; in the same trial efficacy was also shown against severe RSV-LRTD and RSV-ARI.

Differences in baseline characteristics, including body mass index, frailty status, and smoking status, were observed between the Chinese and non-Chinese populations, reflecting inherent demographic and lifestyle differences across regions. Although these factors may influence immune responses to vaccination (21, 22), available evidence indicates that the immunogenicity of the adjuvanted RSVPreF3 vaccine is consistent across key subgroups, including frailty and age categories (23). These differences are therefore likely to have a limited impact on the comparability of immune responses observed in this study. Furthermore, the study was designed and conducted in alignment with Chinese regulatory guidance for immunobridging clinical trials which focuses on demonstrating comparability of immune responses between populations rather than complete baseline homogeneity. Therefore, the immunogenicity data support the extrapolation of adjuvanted RSVPreF3 efficacy to Chinese adults aged ≥60 years and support use of the vaccine in this population. The efficacy observed in the AReSVi-006 trial was also confirmed by real-world data, with several studies showing vaccine effectiveness of 1 dose of adjuvanted RSVPreF3 against RSV-associated hospitalizations in older adults, maintained over 2 consecutive RSV seasons (8). In addition, a recent study characterized RSV F amino acid variations (that could potentially impact vaccine efficacy) in RSV isolates from the AReSVi-006 trial and in strains circulating worldwide (including in China) and assessed vaccine efficacy against predominant strains in the trial. The results supported a broad efficacy of adjuvanted RSVPreF3 across globally circulating RSV strains (24). Moreover, none of the few RT-PCR-confirmed RSV-ARI and RSV-LRTD episodes observed during the surveillance period occurred in vaccinated participants during the current study.

In Chinese participants, adjuvanted RSVPreF3 was more reactogenic than placebo; however, most events were of mild-to-moderate intensity and were transient. The frequency, severity, and duration of solicited administration-site and systemic events were consistent with the known reactogenicity profile of the vaccine (10, 19, 20, 25). The most frequently reported solicited administration-site and systemic events were pain and fatigue, respectively, similar to observations from the AReSVi-006 trial (10); however, reporting rates tended to be lower in the Chinese population than in the global population of the phase 3 pivotal trial (41.7% versus 60.9% for pain and 13.6% versus 33.6% for fatigue).

The incidence of unsolicited AEs reported by vaccine and placebo recipients in China was similar, consistent with previously reported safety data in the global population (10, 20). The frequency of SAEs and pIMDs reported up to study end was low and similar between the Chinese and non-Chinese participants receiving adjuvanted RSVPreF3. Events of atrial fibrillation were rare. There were no reported events of Guillain-Barré syndrome or acute disseminated encephalomyelitis.

The results of this study supported submission of the vaccine for regulatory review in China for its intended use in adults ≥60 years of age. Vaccine approval would cover an unmet medical need, as no RSV-prevention products are currently available in mainland China for this age group. China’s rapidly aging population further heightens the need for effective RSV prevention. The proportion of adults aged ≥60 years is rising steadily, reaching around 20% of the population in 2022, and is projected to reach 28% by 2040 (26, 27). As the population ages, the burden of chronic conditions also rises (27), including those that increase the risk of severe RSV outcomes, such as chronic obstructive pulmonary disease, congestive heart failure, or diabetes (28–30). Taken together, the demographic shift and high prevalence of comorbidities underscore the substantial risk faced by older adults in China and reinforce the need for an RSV vaccine to protect this growing population segment. Interest in RSV vaccination is also high: a recent cross-sectional survey conducted among adults ≥60 years of age attending community health centers from Shenzhen reported that 60.1% of study participants intended to receive the vaccine if fully subsidized (9).

Our study has several limitations. First, the inclusion criteria focused on medically stable adults, which excluded other groups at increased risk of RSV disease (such as immunocompromised individuals) and may limit the generalizability of the findings. Second, the study was not powered to detect very rare AEs, and this limitation should be considered when interpreting safety findings. Finally, the surveillance period and sample size for ARI monitoring were limited, restricting the ability to fully characterize RSV clinical outcomes over time.

## 5 Conclusions

Adjuvanted RSVPreF3 elicited humoral immune responses to RSV-A and RSV-B that were similar between the Chinese and overseas participants, meeting all co-primary immunobridging criteria and demonstrating non-inferiority in terms of GMT ratios and SRR differences in the Chinese cohort. Although neutralizing antibody titers declined by 6 months post-vaccination, levels remained above baseline. The vaccine was well tolerated when administered as a single dose in Chinese adults aged ≥60 years, with no safety signals observed and a safety profile aligned with existing data. Taken together, these findings support the generalizability of vaccine immunogenicity and safety and the extrapolation of global efficacy to the Chinese older adult population and indicate a favorable overall benefit–risk profile.

## Author’s note

*Arexvy* and AS01 are trademarks owned by or licensed to the GSK group of companies. *Abrysvo* is a trademark of Pfizer.

## Data Availability

GSK makes available anonymized individual participant data and associated documents from interventional clinical studies which evaluate medicines, upon approval of proposals submitted to www.clinicalstudydatarequest.com. To access data for other types of GSK sponsored research, for study documents without patient-level data, and for clinical studies not listed, please submit an enquiry via the website.

## The RSV-21 study group

Jing Qiu, Juan Li, Yuting Liao, Zhi Li, and Zhuoying Huang (Shanghai Municipal Center for Disease Control and Prevention), Chunhua Lu (Sijing Township Community Health Service Center); Yeli Zhu (Chedun Township Community Health Service Center); Huijuan Jin (Nanxiang Township Community Health Service Center); Xueping Chen (Waigang Township Community Health Service Center); Huiying Du (Shiquan Street Community Health Service Center); Siwei Xia (Ganquan Street Community Health Service Center); Ling Ni (Qibao Community Health Service Center); Yan Lu (Xinzhuang Community Health Service Center); Haiyan Mao (Jiangchuan Community Health Service Center); Ye Sun (Meilong Community Health Service Center); Liqin Xu (Yanghang Township Community Health Service Center); Dongdong Zhou (Datuan Community Health Service Center); Mariaviviana Bennati, Elvira Maria Leon Gomez, Leo Deng, and Dan Lin (GSK).
