# Supplementary material for: Immunogenicity and safety of a single dose of adjuvanted RSVPreF3 vaccine in adults ≥60 years of age in China: results from a phase 3 immunobridging trial
Source: Front Immunol. 2026 Sep 3;17:1894773. doi: 10.3389/fimmu.2026.1894773 (PMC13582388; doi:10.3389/fimmu.2026.1894773)
Supplement: Supplementary file 1 [file DataSheet1.docx]

# *Supplementary material*

# Text S1. Inclusion and exclusion criteria in the study

## Inclusion criteria

All participants had to satisfy all the following criteria at study entry:

- Adult male or female of ≥60 years of age at the time of study intervention administration, who live in the community dwelling.
- Participants who, in the opinion of the investigator, could and would comply with the requirements of the protocol (e.g., completion of the diary cards, attend regular phone calls/study site visits, perform self-swabbing [study participants in China only], ability to access and utilize a phone or other electronic communications).
- Participants who were medically stable in the opinion of the investigator at the time of vaccination. Participants with chronic stable medical conditions with or without specific treatment, such as diabetes, hypertension or cardiac disease, were allowed to participate in this study if considered by the investigator as medically stable.
- Written or witnessed informed consent obtained from the participant (participant had to be able to understand the informed consent) prior to performance of any study specific procedure.

## Exclusion criteria

Participants were excluded from the study if any of the following criteria applied:

- History of any reaction or hypersensitivity likely to be exacerbated by any component of the study intervention(s).
- Any clinical conditions for which serum samples would be prohibited for transfer to local central laboratory for testing. These clinical conditions included hepatitis B, hepatitis C, HIV and syphilis based on medical history and physical examination (all participants), and laboratory screening tests (non-Chinese participants).
- Any confirmed or suspected immunosuppressive or immunodeficient condition resulting from disease (e.g., current malignancy, human immunodeficiency virus) or immunosuppressive/cytotoxic therapy (e.g., medication used during cancer chemotherapy, organ transplantation, or to treat autoimmune disorders), based on medical history and physical examination (no laboratory testing required).
- Any history of dementia or any medical condition that moderately or severely impairs cognition.
- Recurrent history or uncontrolled neurological disorders or seizures. Participants with medically controlled active or chronic neurological diseases could be enrolled in the study as per investigator assessment, provided that their condition allowed them to comply with the requirements of the protocol (e.g., complete the the diary cards, attend regular phone calls/study site visits, perform self-swabbing [study participants in China only]).
- Significant underlying illness that in the opinion of the investigator would be expected to prevent completion of the study (e.g., life-threatening disease likely to limit survival to less than 1 year).
- Serious or unstable chronic illness.
- Any other clinical condition that, in the opinion of the investigator, might pose additional risk to the participant due to participation in the study.
- Previous vaccination with any respiratory syncytial virus vaccine.
- Use of any investigational or non-registered product (drug, vaccine or invasive medical device) other than the study intervention(s) during the period beginning 30 days before the dose of study intervention(s), or their planned use during the study period.
- Planned or actual administration of a vaccine not foreseen by the study protocol in the period starting 30 days before and ending 30 days after study intervention administration, with the exception of COVID-19 and inactivated/subunit influenza vaccines which could be administered up to 14 days before or from 14 days after each study intervention.
- Administration of long-acting immune-modifying drugs or planned administration at any time during the study period (e.g., infliximab).
- Administration of immunoglobulins and/or any blood products or plasma derivatives during the period starting 90 days before the study intervention administration or planned administration during the study period.
- Chronic administration (defined as more than 14 consecutive days in total) of immunosuppressants or other immune-modifying drugs during the period starting 90 days prior to the study intervention administration or planned administration during the study period. For corticosteroids, this meant prednisone ≥20 mg/day, or equivalent. Inhaled and topical steroids were allowed.
- Concurrently participating in another clinical study, at any time during the study period, in which the participant was or would be exposed to an investigational or a non-investigational intervention (drug/invasive medical device).
- History of chronic alcohol consumption and/or drug abuse as deemed by the investigator to render the potential participant unable/unlikely to provide accurate safety reports or comply with study procedures.
- Bedridden participants.
- Planned move during the study conduct that prohibited participation until study end.
- Participation of any study personnel or their immediate dependents, family, or household members.

# Supplementary Table 1. Case definitions for ARI and LRTD

| **Endpoint** | **Case definition** | | |
| --- | --- | --- | --- |
| ARI  (trigger for swabbing) | Presence of:   - at least 2 respiratory symptoms/signs for at least 24 hours   OR   - at least 1 respiratory symptom/sign + 1 systemic symptom/sign for at least 24 hours | | |
|  | Respiratory symptoms and signs  - Nasal congestion/rhinorrhea  - Sore throat  - New or increased sputum  - New or increased cough  - New or increased dyspnea (shortness of breath)  - New or increased wheezing^b^  - New or increased crackles/ronchi^c^ based on chest auscultation  - Respiratory rate ≥20 respirations/min^c^  - Low or decreased oxygen saturation (O_2_ saturation <95% or ≤90% if pre-season baseline is <95%)^c^  - Need for oxygen supplementation^c^ | | Systemic symptoms and signs  - Temperature ≥38.0°C/feverishness^a^  - Fatigue  - Body aches  - Headache  - Decreased appetite |
| RT-PCR-confirmed RSV-ARI^d^ | An event meeting the case definition of ARI with at least 1 RSV-positive swab detected by RT-PCR | | |
| LRTD | Presence of:   - at least 2 lower respiratory symptoms/signs for at least 24 hours including at least 1 lower respiratory sign   OR   - at least 3 lower respiratory symptoms for at least 24 hours | | |
|  | Lower respiratory symptoms  - New or increased sputum  - New or increased cough  - New or increased dyspnea (shortness of breath) | Lower respiratory signs  - New or increased wheezing^b^  - New or increased crackles/ronchi^c^ based on chest auscultation  - Respiratory rate ≥20 respirations/min^c^  - Low or decreased oxygen saturation (O_2_ saturation <95% or ≤90% if pre-season baseline is <95%)^c^  - Need for oxygen supplementation^c^ | |
| RT-PCR-confirmed RSV-LRTD^d^ | An event meeting the case definition of LRTD with at least 1 RSV-positive swab detected by RT-PCR | | |
| RT-PCR-confirmed severe RSV-LRTD – Definition 1 “Clinical symptomology”^d^ | Presence of a LRTD with the following criteria:   - at least 2 lower respiratory signs   AND   - with at least 1 RSV-positive swab detected by RT-PCR | | |
|  | Lower respiratory signs  - New or increased wheezing^b^  - New or increased crackles/ronchi^c^ based on chest auscultation  - Respiratory rate ≥20 respirations/min^3^  - Low or decreased oxygen saturation (O_2_ saturation <95% or ≤90% if pre-season baseline is <95%)^c^  - Need for oxygen supplementation^c^ | | |
| RT-PCR-confirmed severe RSV-LRTD – Definition 2 “Supportive therapy”^d^ | Presence of a LRTD with at least one of the following criteria^e^:   - Need for oxygen supplementation^c^ - Need for positive airway pressure therapy (e.g. CPAP) - Need for other types of mechanical ventilation   AND   - with at least 1 RSV-positive swab detected by RT-PCR | | |

ARI, acute respiratory illness; CPAP, continuous positive airway pressure; LRTD, lower respiratory tract disease; RSV, respiratory syncytial virus; RT-PCR, reverse transcription polymerase chain reaction.

Notes:	^a^Feverishness was defined as the feeling of having fever without objective measurement; ^b^reported by study participant or investigator; ^c^reported by investigator; ^d^throat and/or nasal swab samples collected at ARI visits for RT-PCR testing were collected within 6 days after ARI onset (i.e., up to Day 7); ^e^in case the participant was already receiving any of these for treating/controlling any pre-existing condition, any significant change or adaptation in the used therapy was taken into account.

# Supplementary Table 2. Participant characteristics at baseline (exposed set)

|  | **RSVPreF3_China**  **N=1199** | **Placebo_China**  **N=601** | **RSVPreF3_Overseas**  **N=820** | **Total**  **N=2620** |
| --- | --- | --- | --- | --- |
| Mean age ±SD, years | 69.8±6.6 | 69.9±6.7 | 70.5±6.8 | 70.1±6.7 |
| Age category, n (%) |  |  |  |  |
| 60–69 years | 612 (51.0) | 314 (52.2) | 397 (48.4) | 1323 (50.5) |
| 70–79 years | 479 (39.9) | 223 (37.1) | 324 (39.5) | 1026 (39.2) |
| ≥80 years | 108 (9.0) | 64 (10.6) | 99 (12.1) | 271 (10.3) |
| Female, n (%) | 618 (51.5) | 304 (50.6) | 439 (53.5) | 1361 (51.9) |
| Race, n (%) |  |  |  |  |
| Asian - Central/South Asian Heritage | 0 (0.0) | 0 (0.0) | 9 (1.1) | 9 (0.3) |
| Asian - Chinese Heritage | 1199 (100) | 601 (100) | 2 (0.2) | 1802 (68.8) |
| Asian - Korean Heritage | 0 (0.0) | 0 (0.0) | 60 (7.3) | 60 (2.3) |
| Asian - Japanese Heritage | 0 (0.0) | 0 (0.0) | 31 (3.8) | 31 (1.2) |
| White-Caucasian/European Heritage | 0 (0.0) | 0 (0.0) | 707 (86.2) | 707 (27.0) |
| Other | 0 (0.0) | 0 (0.0) | 11 (1.3) | 11 (0.4) |
| Mean BMI (±SD), kg/m² | 25.1±3.3 | 25.1±3.1 | 27.6±4.8 | 25.9±3.9 |
| Frailty status, n (%) |  |  |  |  |
| Frail | 13 (1.1) | 5 (0.8) | 2 (0.2) | 20 (0.8) |
| Pre-frail | 614 (51.2) | 300 (49.9) | 214 (26.1) | 1128 (43.1) |
| Fit | 572 (47.7) | 296 (49.3) | 604 (73.7) | 1472 (56.2) |
| Smoker status for tobacco |  |  |  |  |
| Current smoker | 211 (17.6) | 110 (18.3) | 88 (10.7) | 409 (15.6) |
| Former smoker | 167 (13.9) | 80 (13.3) | 317 (38.7) | 564 (21.5) |
| Never smoked | 821 (68.5) | 411 (68.4) | 415 (50.6) | 1647 (62.9) |
| Smoker status for e-cigarettes |  |  |  |  |
| Current smoker | 0 (0.0) | 0 (0.0) | 0 (0.0) | 0 (0.0) |
| Former smoker | 0 (0.0) | 0 (0.0) | 5 (0.6) | 5 (0.2) |
| Never smoked | 1199 (100) | 601 (100) | 802 (97.8) | 2602 (99.3) |
| Missing | 0 (0.0) | 0 (0.0) | 13 (1.6) | 13 (0.5) |

BMI, body mass index; n (%), number (percentage) of participants in each category; N, number of participants in each group; SD, standard deviation.

Notes:	Other included American Indian or Alaska Native, Asian - South East Asian Heritage, and Black or African American.

# Supplementary Table 3. Summary of results of non-inferiority objectives for the GMT ratio and the SRR difference (RSVPreF3_Overseas group versus RSVPreF3_China group) at 1 month post-vaccination (per protocol set)

| **Endpoint** | **Success criteria** | **GMT ratio (95% CI)** | **SRR difference (95% CI)** | **Success** |
| --- | --- | --- | --- | --- |
| RSV-A neutralization titers (ED60) | UL of the 95% CI: ≤1.5 for GMT ratio and ≤10% for SRR difference | 1.11 (1.04–1.20) | -0.39 (-4.29–3.40) | Yes |
| RSV-B neutralization titers (ED60) |  | 1.03 (0.95–1.10) | 0.75 (-3.48–4.89) | Yes |

CI, confidence interval; ED60, estimated dilution 60; GMT, geometric mean titer; RSV, respiratory syncytial virus; SRR, seroresponse rate; UL, upper limit.

Notes:	The group ratio of adjusted GMT was performed using an ANCOVA model applied to the log_10_-transformed titers, which included the group as fixed effect and the pre-dose log_10_-transformed titers as covariate.

The SRR was defined as the proportion of participants having ≥4-fold increase in neutralizing titers (1 month post-vaccination [Day 31] over pre-vaccination [Day 1]).

# Supplementary Table 4. Ratio of adjusted GMCs and SRRs difference for RSV-A and RSV-B neutralizing concentrations (IU/mL) between the RSVPreF3_Overseas group and the RSVPreF3_China group at Day 31 (per protocol set)

| **Endpoint** | **RSVPreF3_Overseas** | |  | **RSVPreF3_China** | |  | **RSVPreF3_Overseas versus RSVPreF3_China** |  |
| --- | --- | --- | --- | --- | --- | --- | --- | --- |
|  | **N** | **value (95% CI)** |  | **N** | **value (95% CI)** |  | **value (95% CI)** | |
| RSV-A |  |  |  |  |  |  |  | |
| Adjusted GMC | 757 | 13573.3 (12811.6–14380.2) |  | 1175 | 12403.1 (11841.7–12991.0) |  | 1.09 (1.02–1.18) | |
| SRR |  | 76.9 (73.7–79.8) |  |  | 76.8 (74.2–79.2) |  | 0.12 (-3.79–3.92) | |
| RSV-B |  |  |  |  |  |  |  | |
| Adjusted GMC | 757 | 10066.9 (9479.1–10691.2) |  | 1175 | 10559.0 (10061.3–11081.3) |  | 0.95 (0.88–1.03) | |
| SRR |  | 69.1 (65.7–72.4) |  |  | 71.4 (68.7–74.0) |  | -2.32 (-6.53–1.84) | |

CI, confidence interval; GMC, geometric mean concentration; IU, International Units; N, number of participants with available results; RSV, respiratory syncytial virus; SRR, seroresponse rate.

Notes:	The group ratio of adjusted GMC was performed using an ANCOVA model applied to the log_10_-transformed concentrations, which included the group as fixed effect and the pre-dose log_10_-transformed concentrations as covariate.

The SRR was defined as the proportion of participants having ≥4-fold increase in neutralizing concentrations (1 month post-vaccination [Day 31] over pre-vaccination [Day 1]).

# Supplementary Table 5. Ratio of adjusted GMTs/GMCs and SRRs difference for RSV-A and RSV-B neutralizing titers (ED60)/concentrations (IU/mL) between the RSVPreF3_Overseas group and the RSVPreF3_China group at Day 31 (exposed set)

| **Endpoint** | **RSVPreF3_Overseas** | |  | **RSVPreF3_China** | |  | **RSVPreF3_Overseas versus RSVPreF3_China** |  |
| --- | --- | --- | --- | --- | --- | --- | --- | --- |
|  | **N** | **value (95% CI)** |  | **N** | **value (95% CI)** |  | **value (95% CI)** | |
| RSV-A |  |  |  |  |  |  |  | |
| Adjusted GMT | 815 | 6325.5 (5981.4–6689.3) |  | 1190 | 5660.1 (5404.3–5928.0) |  | 1.12 (1.04–1.20) | |
| SRR |  | 76.8 (73.8–79.7) |  |  | 77.1 (74.6–79.4) |  | -0.25 (-4.05–3.47) | |
| Adjusted GMC |  | 13555.5 (12820.7–14332.4) |  |  | 12366.1 (11809.1–12949.4) |  | 1.10 (1.02–1.18) | |
| SRR |  | 76.8 (73.8–79.7) |  |  | 76.6 (74.0–78.9) |  | 0.26 (-3.55–3.99) | |
| RSV-B |  |  |  |  |  |  |  | |
| Adjusted GMT | 815 | 8332.4 (7882.8–8807.7) |  | 1190 | 8067.3 (7705.3–8446.3) |  | 1.03 (0.96–1.11) | |
| SRR |  | 70.4 (67.2–73.5) |  |  | 69.3 (66.6–71.9) |  | 1.10 (-3.01–5.16) | |
| Adjusted GMC |  | 10121.0 (9546.6–10730.1) |  |  | 10556.0 (10057.8–11079.0) |  | 0.96 (0.89–1.03) | |
| SRR |  | 68.8 (65.5–72.0) |  |  | 71.2 (68.5–73.7) |  | -2.34 (-6.46–1.72) | |

CI, confidence interval; ED60, estimated dilution 60; GMC, geometric mean concentration; GMT, geometric mean titer; IU, International Units; N, number of participants with available results; RSV, respiratory syncytial virus; SRR, seroresponse rate.

Notes:	The group ratio of adjusted GMT/GMC was performed using an ANCOVA model applied to the log_10_-transformed titers/concentrations, which included the group as fixed effect and the pre-dose log_10_-transformed titers/concentrations as covariate.

The SRR was defined as the proportion of participants having ≥4-fold increase in neutralizing titers/concentrations (1 month post-vaccination [Day 31] over pre-vaccination [Day 1]).

# Supplementary Table 6. Number and percentage of participants with RSV-A and RSV-B neutralizing titers/concentrations equal to or above the assay cut-off, GMT (ED60)/GMC (IU/mL), SRR, and MGI in each group (per protocol set)

|  |  | **value (95% CI)** | | |
| --- | --- | --- | --- | --- |
| **Timepoint** | **Endpoint** | **RSVPreF3_China** | **Placebo_China** | **RSVPreF3_Overseas** |
| RSV-A |  |  |  |  |
| Results presented in ED60 | | | | |
| Day 1 | N | 1191 | 598 | 816 |
|  | ≥23 ED60 | 100 (99.7–100) | 100 (99.4–100) | 100 (99.5–100) |
|  | GMT | 703.3 (674.0–733.9) | 642.7 (606.7–680.8) | 825.8 (776.6–878.1) |
| Day 31 | N | 1175 | 590 | 757 |
|  | ≥23 ED60 | 100 (99.7–100) | 100 (99.4–100) | 100 (99.5–100) |
|  | GMT | 5523.2 (5254.7–5805.5) | 637.9 (600.1–678.1) | 6604.5 (6194.4–7041.7) |
|  | MGI | 7.89 (7.49–8.32) | 0.99 (0.95–1.03) | 8.04 (7.51–8.60) |
|  | SRR | 77.3 (74.8–79.6) | 0.5 (0.1–1.5) | 76.9 (73.7–79.8) |
| Month 6 | N | 1157 | 584 | 786 |
|  | ≥23 ED60 | 100 (99.7–100) | 100 (99.4–100) | 100 (99.5–100) |
|  | GMT | 2283.4 (2173.7–2398.6) | 615.0 (582.6–649.1) | 2395.6 (2253.9–2546.3) |
|  | MGI | 3.27 (3.12–3.42) | 0.96 (0.92–0.99) | 2.89 (2.72–3.07) |
|  | SRR | 36.7 (33.9–39.6) | 0.9 (0.3–2.0) | 34.0 (30.7–37.4) |
| Results presented in IU/mL | | | | |
| Day 1 | N | 1191 | 598 | 816 |
|  | ≥57 mIU/mL | 100 (99.7–100) | 100 (99.4–100) | 100 (99.5–100) |
|  | GMC | 1567.1 (1504.1–1632.7) | 1447.0 (1367.4–1531.4) | 1804.2 (1699.4–1915.4) |
| Day 31 | N | 1175 | 590 | 757 |
|  | ≥57 mIU/mL | 100 (99.7–100) | 100 (99.4–100) | 100 (99.5–100) |
|  | GMC | 12109.0 (11524.4–12723.2) | 1469.8 (1383.3–1561.7) | 14088.4 (13217.4–15016.8) |
|  | MGI | 7.77 (7.38–8.18) | 1.01 (0.97–1.06) | 7.86 (7.34–8.41) |
|  | SRR | 76.8 (74.2–79.2) | 0.7 (0.2–1.7) | 76.9 (73.7–79.8) |
| Month 6 | N | 1157 | 584 | 786 |
|  | ≥57 mIU/mL | 100 (99.7–100) | 100 (99.4–100) | 100 (99.5–100) |
|  | GMC | 6216.3 (5917.9–6529.8) | 1647.7 (1557.9–1742.7) | 6677.6 (6274.4–7106.7) |
|  | MGI | 4.00 (3.81–4.19) | 1.14 (1.09–1.19) | 3.69 (3.47–3.92) |
|  | SRR | 47.8 (44.9–50.7) | 1.0 (0.4–2.2) | 44.3 (40.8–47.8) |
| RSV-B |  |  |  |  |
| Results presented in ED60 | | | | |
| Day 1 | N | 1191 | 598 | 816 |
|  | ≥30 ED60 | 100 (99.7–100) | 100 (99.4–100) | 100 (99.5–100) |
|  | GMT | 1264.9 (1209.8–1322.6) | 1155.2 (1086.6–1228.2) | 1274.2 (1194.6–1359.0) |
| Day 31 | N | 1175 | 590 | 757 |
|  | ≥30 ED60 | 100 (99.7–100) | 100 (99.4–100) | 100 (99.5–100) |
|  | GMT | 8073.2 (7680.5–8485.9) | 1092.1 (1027.6–1160.7) | 8286.2 (7766.3–8840.9) |
|  | MGI | 6.40 (6.08–6.73) | 0.94 (0.91–0.98) | 6.57 (6.12–7.04) |
|  | SRR | 69.5 (66.8–72.2) | 0.5 (0.1–1.5) | 70.3 (66.9–73.5) |
| Month 6 | N | 1158 | 584 | 786 |
|  | ≥30 ED60 | 100 (99.7–100) | 100 (99.4–100) | 100 (99.5–100) |
|  | GMT | 3931.5 (3742.4–4130.1) | 1135.6 (1065.3–1210.6) | 3785.0 (3557.0–4027.6) |
|  | MGI | 3.12 (2.98–3.27) | 0.98 (0.94–1.03) | 2.96 (2.78–3.14) |
|  | SRR | 36.7 (33.9–39.6) | 1.0 (0.4–2.2) | 33.5 (30.2–36.9) |
| Results presented in IU/mL | | | | |
| Day 1 | N | 1191 | 598 | 816 |
|  | ≥44 IU/mL | 100 (99.7–100) | 100 (99.4–100) | 100 (99.5–100) |
|  | GMC | 1632.3 (1561.7–1706.0) | 1477.9 (1390.3–1571.0) | 1519.9 (1425.7–1620.2) |
| Day 31 | N | 1175 | 590 | 757 |
|  | ≥44 IU/mL | 100 (99.7–100) | 100 (99.4–100) | 100 (99.5–100) |
|  | GMC | 10710.7 (10167.7–11282.8) | 1451.0 (1363.4–1544.2) | 9846.4 (9195.9–10543.0) |
|  | MGI | 6.58 (6.24–6.94) | 0.98 (0.94–1.02) | 6.54 (6.09–7.03) |
|  | SRR | 71.4 (68.7–74.0) | 1.0 (0.4–2.2) | 69.1 (65.7–72.4) |
| Month 6 | N | 1158 | 584 | 786 |
|  | ≥44 IU/mL | 100 (99.7–100) | 100 (99.4–100) | 100 (99.5–100) |
|  | GMC | 5895.1 (5605.4–6199.7) | 1679.5 (1574.0–1792.1) | 5824.5 (5458.2–6215.3) |
|  | MGI | 3.62 (3.46–3.79) | 1.13 (1.09–1.18) | 3.81 (3.58–4.05) |
|  | SRR | 42.8 (40.0–45.7) | 1.4 (0.6–2.7) | 45.7 (42.2–49.2) |

CI, confidence interval; ED60, estimated dilution 60; GMC, geometric mean concentration; GMT, geometric mean titer; IU, International Units; MGI, mean geometric increase from Day 1; N, number of participants with available results; RSV, respiratory syncytial virus; SRR, seroresponse rate.

Note:	The SRR was defined as the proportion of participants having ≥4-fold increase in neutralizing titers/concentrations (1 month post-vaccination [Day 31] over pre-vaccination [Day 1]).

# Supplementary Table 7. Number and percentage of participants with solicited administration-site and systemic events (exposed set)

|  | **RSVPreF3_China** | |  | **Placebo_China** | |
| --- | --- | --- | --- | --- | --- |
|  | **n** | **% (95% CI)** |  | **n** | **% (95% CI)** |
| Administration-site events | N=1193 | |  | N=599 | |
| Erythema |  |  |  |  |  |
| Any | 88 | 7.4 (6.0–9.0) |  | 3 | 0.5 (0.1–1.5) |
| Grade 3 | 3 | 0.3 (0.1–0.7) |  | 0 | 0.0 (0.0–0.6) |
| Medically attended event | 0 | 0.0 (0.0–0.3) |  | 0 | 0.0 (0.0–0.6) |
| Erythema - China grading |  |  |  |  |  |
| Any | 88 | 7.4 (6.0–9.0) |  | 3 | 0.5 (0.1–1.5) |
| Grade 3 | 3 | 0.3 (0.1–0.7) |  | 0 | 0.0 (0.0–0.6) |
| Medically attended event | 0 | 0.0 (0.0–0.3) |  | 0 | 0.0 (0.0–0.6) |
| Pain |  |  |  |  |  |
| Any | 497 | 41.7 (38.8–44.5) |  | 25 | 4.2 (2.7–6.1) |
| Grade 3 | 6 | 0.5 (0.2–1.1) |  | 0 | 0.0 (0.0–0.6) |
| Medically attended event | 0 | 0.0 (0.0–0.3) |  | 0 | 0.0 (0.0–0.6) |
| Swelling |  |  |  |  |  |
| Any | 82 | 6.9 (5.5–8.5) |  | 0 | 0.0 (0.0–0.6) |
| Grade 3 | 2 | 0.2 (0.0–0.6) |  | 0 | 0.0 (0.0–0.6) |
| Medically attended event | 0 | 0.0 (0.0–0.3) |  | 0 | 0.0 (0.0–0.6) |
| Swelling - China grading |  |  |  |  |  |
| Any | 81 | 6.8 (5.4–8.4) |  | 0 | 0.0 (0.0–0.6) |
| Grade 3 | 2 | 0.2 (0.0–0.6) |  | 0 | 0.0 (0.0–0.6) |
| Medically attended event | 0 | 0.0 (0.0–0.3) |  | 0 | 0.0 (0.0–0.6) |
| Systemic events | N=1193 | |  | N=600 | |
| Arthralgia |  |  |  |  |  |
| Any | 67 | 5.6 (4.4–7.1) |  | 11 | 1.8 (0.9–3.3) |
| Grade 3 | 4 | 0.3 (0.1–0.9) |  | 0 | 0.0 (0.0–0.6) |
| Medically attended event | 1 | 0.1 (0.0–0.5) |  | 2 | 0.3 (0.0–1.2) |
| Fatigue |  |  |  |  |  |
| Any | 162 | 13.6 (11.7–15.7) |  | 19 | 3.2 (1.9–4.9) |
| Grade 3 | 2 | 0.2 (0.0–0.6) |  | 0 | 0.0 (0.0–0.6) |
| Medically attended event | 1 | 0.1 (0.0–0.5) |  | 4 | 0.7 (0.2–1.7) |
| Fever (°C) |  |  |  |  |  |
| ≥38.0 | 30 | 2.5 (1.7–3.6) |  | 3 | 0.5 (0.1–1.5) |
| >38.5 | 8 | 0.7 (0.3–1.3) |  | 2 | 0.3 (0.0–1.2) |
| >39.0 | 2 | 0.2 (0.0–0.6) |  | 1 | 0.2 (0.0–0.9) |
| >39.5 | 0 | 0.0 (0.0–0.3) |  | 1 | 0.2 (0.0–0.9) |
| >40.0 | 0 | 0.0 (0.0–0.3) |  | 0 | 0.0 (0.0–0.6) |
| Medically attended event | 4 | 0.3 (0.1–0.9) |  | 5 | 0.8 (0.3–1.9) |
| Fever (°C) – China grading |  |  |  |  |  |
| ≥37.3 | 128 | 10.7 (9.0–12.6) |  | 10 | 1.7 (0.8–3.0) |
| ≥38.0 | 30 | 2.5 (1.7–3.6) |  | 3 | 0.5 (0.1–1.5) |
| ≥38.5 | 8 | 0.7 (0.3–1.3) |  | 2 | 0.3 (0.0–1.2) |
| ≥39.0 | 2 | 0.2 (0.0–0.6) |  | 1 | 0.2 (0.0–0.9) |
| ≥39.5 | 0 | 0.0 (0.0–0.3) |  | 1 | 0.2 (0.0–0.9) |
| ≥39.5 persisting >3 days | 0 | 0.0 (0.0–0.3) |  | 0 | 0.0 (0.0–0.6) |
| Medically attended event | 4 | 0.3 (0.1–0.9) |  | 5 | 0.8 (0.3–1.9) |
| Headache |  |  |  |  |  |
| Any | 93 | 7.8 (6.3–9.5) |  | 14 | 2.3 (1.3–3.9) |
| Grade 3 | 2 | 0.2 (0.0–0.6) |  | 0 | 0.0 (0.0–0.6) |
| Medically attended event | 1 | 0.1 (0.0–0.5) |  | 3 | 0.5 (0.1–1.5) |
| Myalgia |  |  |  |  |  |
| Any | 123 | 10.3 (8.6–12.2) |  | 12 | 2.0 (1.0–3.5) |
| Grade 3 | 3 | 0.3 (0.1–0.7) |  | 0 | 0.0 (0.0–0.6) |
| Medically attended event | 0 | 0.0 (0.0–0.3) |  | 1 | 0.2 (0.0–0.9) |

CI, confidence interval; n (%), number (percentage) of participants with at least 1 event; N, total number of participants with diary cards.

Note:	Grade 3 events were defined as significant pain at rest, preventing normal everyday activities for pain, a diameter >100 mm for swelling and erythema, an axillary temperature >39.0°C for fever, and as preventing normal everyday activities for the other events. According to the Chinese grading scale, Grade 3 erythema or swelling was defined as a diameter ≥100 mm, Grade 3 fever was as an axillary temperature ≥38.5°C, and Grade 4 fever was defined as an axillary temperature ≥39.5°C and lasting more than 3 days.
